# Transforming Caring for Dependent Older People: A New Approach for Integrated Socio‐Healthcare

**DOI:** 10.1111/scs.70132

**Published:** 2025-10-08

**Authors:** Ana‐María Porcel‐Gálvez, Regina Allande‐Cussó, Marta Lima‐Serrano

**Affiliations:** ^1^ Nursing Department, Faculty of Nursing, Physiotherapy and Podiatry University of Seville Seville Spain; ^2^ Research Group CTS 1050 Complex Care, Chronicity and Health Outcomes Institute of Biomedicine of Seville (IBiS) Seville Spain; ^3^ Research Group CTS 969 Care Innovation and Health Determinants Institute of Biomedicine of Seville (IBiS) Seville Spain

**Keywords:** care dependency, digital health solutions, health equity, integrated care, person‐centred care, social inclusion

## Abstract

**Background:**

The increasing ageing population and growing socioeconomic disparities in the Mediterranean region pose significant challenges to the provision of integrated socio‐healthcare services. Current models often fail to address the complex needs of dependent older adults and those at risk of social exclusion, highlighting the need for a sustainable, person‐centred approach.

**Objectives:**

This study aims to develop and theoretically ground the TEC‐MED framework, a novel model for integrated socio‐health care that ensures holistic, ethical, and culturally responsive care for vulnerable older populations.

**Methods:**

A concept analysis following Walker and Avant's methodology was conducted, informed by prior empirical research. The framework was designed based on the nursing metaparadigm, incorporating interdisciplinary collaboration, digital health solutions, and policy integration at micro, meso, and macro levels.

**Results:**

The TEC‐MED framework consists of six key dimensions (person, socio‐health professionals, care context, service provision, governance, and financing) and five transversal values (quality research, gender equity, social inclusion, ethics, and transcultural care). The model enhances interdisciplinary coordination, digital innovation, and patient‐centered care, addressing disparities in access and service fragmentation. Case studies demonstrate its applicability in diverse healthcare settings.

**Conclusion:**

The TEC‐MED framework offers an innovative and sustainable solution for improving socio‐healthcare integration, ensuring dignified and inclusive care for dependent older adults. Its implementation has significant implications for clinical practice, policymaking, and professional training, reinforcing the humanisation of care while promoting health equity and system sustainability.

## 1 Introduction

Population ageing and the rising prevalence of chronic diseases pose critical challenges to socio‐healthcare systems in the Mediterranean region. According to the United Nations [1] and the World Health Organization [2], people over the age of 65 will represent more than 25% of the population in this region by 2050, with life expectancy already exceeding 80 years in several countries. Simultaneously, the old‐age dependency ratio is projected to increase sharply, intensifying pressure on long‐term care systems [3]. This demographic shift is compounded by high levels of economic inequality and social vulnerability: over 20% of older adults in the Mediterranean are at risk of poverty and social exclusion [3]. The growing burden of noncommunicable diseases, such as cardiovascular conditions, diabetes, chronic respiratory diseases, and neurodegenerative disorders, further contributes to the demand for specialised and sustained care [4]. These combined trends underscore the urgent need for integrated and sustainable socio‐healthcare solutions for dependent older adults, particularly those facing geographic, financial, or cultural barriers to access [5].

While care dependency and risk of social exclusion are distinct conditions, they frequently coexist and intersect in older populations, particularly in socioeconomically vulnerable regions [6]. Dependency often leads to reduced autonomy, limited mobility, and increased need for long‐term support, whereas social exclusion may stem from poverty, isolation, lack of access to services, or cultural barriers. However, both conditions share underlying determinants, such as weakened social networks, marginalisation, and health inequities, which result in similar challenges for accessing integrated and person‐centred care [7].

Additionally, healthcare systems in these countries show significant differences in terms of funding, coverage, and quality, limiting the implementation of integrated and personalised care models [1]. These disparities are particularly evident between high‐income countries, such as Spain and Italy, which have established universal healthcare systems, and lower‐income countries, such as Morocco and Egypt, where access to publicly funded services remains limited [8]. Furthermore, projections indicate that public expenditure on ageing‐related healthcare and social services will increase by more than 30% by 2050 if no structural reforms are implemented, further challenging the financial sustainability of current models [9].

The Mediterranean countries share cultural ties, challenges, and needs. In this context, the ENI CBC “Mediterranean Sea Basin Programme” is the largest Cross‐Border Cooperation (CBC) initiative implemented by the EU under the European Neighbourhood Instrument (ENI) 2014–2020. The programme brings together coastal territories from 14 countries to finance innovative, inclusive, and sustainable cooperation for development on both shores of the Mediterranean. The projects are organised into four thematic groups, one of which focuses on social inclusion and the fight against poverty. Within this group, the TEC‐MED project (*Development of a Transcultural Social and Ethical Care Model for Dependent Populations in the Mediterranean Basin*) [10, 11] is anchored. The project aims to develop a transcultural social and ethical care model for vulnerable older populations in the Mediterranean. In alignment with this objective, Spain (Lead Beneficiary [LB]), along with Greece, Italy, Lebanon, Egypt, and Tunisia, is participating partners. This approach effectively addresses the complex needs of dependent older adults, ensuring their dignity, autonomy, and active participation in care processes [12].

The TEC‐MED approach highlights the central role of the nursing metaparadigm [13], structured around the following concepts: ‘Person’, which considers cultural contexts; ‘Environment’, encompassing cultural, social, and economic influences; ‘Health’, which involves vital processes; and ‘Nursing Role’, which pertains to practice [14]. This framework provides a holistic and person‐centred perspective. It places older individuals, their families, and caregivers at the heart of care, ensuring that all socio‐healthcare practices include home care, social networks, or community centres rather than relying solely on residential settings. Socio‐health care services are provided by multidisciplinary teams adopting a universal health perspective, focussing on autonomy of self‐care, well‐being, community participation, and mental health [15]. The project framework is structured across three levels of management: micro, meso, and macro [16] to ensure a comprehensive and sustainable response to the needs of older people dependent on and those at risk of social exclusion, who constitute the target population of the study. Furthermore, the implementation of innovative technological solutions, such as digital health platforms and telemedicine, plays a crucial role in enhancing service delivery and facilitating coordinated care [17, 18, 19].

Among the project's achievements are improved social service coverage for approximately 22,000 people, the signing of 12 agreements between public administrations and key stakeholders to facilitate the coordinated planning and implementation of social services, the participation of 90 social care professionals in pilot social schemes, the development of six action plans for public administrations, and the creation of an online platform for cooperation and partnerships between public institutions and social care actors [10, 11].

The need to research and further develop the TEC‐MED framework also arises from the lack of integrative and sustainable policies in Mediterranean countries. Currently, care systems operate largely in isolation, limiting their ability to provide comprehensive and continuous care, with significant fragmentation between primary, specialised, and social care. This fragmentation directly affects the health outcomes and quality of life of older adults, particularly those living in rural areas or facing poverty [20].

In this context, the TEC‐MED model seeks to transform older people's care in the Mediterranean basin through the integration of socio‐health care services, the adoption of innovative technologies and the implementation of inclusive policies. Its holistic approach, grounded in the nursing metaparadigm and structured across the micro, meso, and macro levels, aims to advance toward a more equitable and sustainable socio‐healthcare model. Although the TEC‐MED project led to the implementation of a preliminary socio‐healthcare model, its structure was primarily based on empirical insights and expert consensus. Building upon this foundation, the present study applies a structured concept analysis to examine, refine, and theoretically validate the model. Accordingly, the objective of this study is to develop and theoretically ground a new framework for integrated socio‐health care for older people with dependency and those at risk of social exclusion, as a necessary step prior to empirical validation. This framework is based on the concept analysis proposed by Walker and Avant [21], which helps clarify and optimise the key elements of integrated care. This process involves identifying antecedents, attributes, model and contrary cases, as well as empirical referents that validate the practical application of the framework, supported by previous research to establish the TEC‐MED framework.

The TEC‐MED framework addresses two overlapping but not identical populations: older adults who are dependent and those at risk of social exclusion. While these groups may present distinct primary needs, such as functional support in the case of dependency and access barriers or marginalisation in the case of social exclusion, they often coexist and share underlying vulnerabilities, such as limited autonomy, reduced access to services, and social isolation. Therefore, the framework has been designed to be adaptable, enabling tailored interventions that respond to the specific risks and needs of each group within a unified and person‐centred model.

## 2 Methods

Concept analysis is a method proposed by Walker and Avant consisting of a thorough study of the descriptions of a concept and its meanings. Concept analysis is a process of explicating a phenomenon to achieve a better understanding and maximum optimisation [21]. According to Walker and Avant, the process of analysing concepts involves eight stages: [1] selecting a concept; [2] determining the aim and purpose of the analysis; [3] identifying the uses of the concept; [4] defining attributes, identifying characteristics that appear repeatedly when it is defined in different sources; [5] identifying a model case, which presents all the identified attributes; [6] identifying related, borderline, and contrary cases, in which the concept is not clearly represented or situations contrary to the model case; [7] identifying the antecedents, or starting situations, and consequences of the concept, understood as effects or outcomes of the concept; and [8] defining the empirical referents that demonstrate the possibility of the phenomenon in real contexts and how to study it [21]. Concept analysis was selected because the TEC‐MED framework required a rigorous theoretical clarification of its attributes, antecedents, and consequences before empirical validation.

The present concept analysis was based on previous field research carried out by the research team (GAP analysis and integrative review) to clarify the definition of the TEC‐MED framework. Considering this information, the team went back and forth on the steps in the analysis of the concept to avoid premature conclusions.

### 2.1 Ethical Considerations

Although concept analysis does not involve direct data collection from human subjects, the present study is grounded in previous empirical research conducted within the TEC‐MED project, which received ethical approval from the relevant committees in each participating country. All data used in the preparatory phases, including interviews and Delphi panels, were collected in compliance with ethical standards, including informed consent, confidentiality, and the voluntary nature of participation. In applying this methodological approach, particular attention was paid to safeguarding the dignity and autonomy of vulnerable older adults, especially in contexts marked by socioeconomic and cultural disparities.

## 3 Results

This section presents the results obtained following the Walker and Avant method [21].

### 3.1 Selecting a Concept

The increasing demand for long‐term care services, coupled with demographic ageing and socioeconomic disparities, has highlighted the need for innovative and sustainable care paradigms for older adults, particularly those at risk of social exclusion in the Mediterranean region [22]. However, current care models may not adequately address the complex needs of this population, necessitating a structured approach to redefine and optimize care provision.

To analyse and understand social care practices for the elderly population facing dependency and social exclusion, a SWOT analysis was conducted in 28 semistructured interviews with key stakeholders at various management levels who were interviewed in Egypt, Greece, Lebanon, Spain and Tunisia [23]. This revealed strengths in legislation, financing, and services, as well as weaknesses in financial resources, personnel training, and organisational coherence. Opportunities included cultural values, legislation, technology, and changing care paradigms, while threats included coordination issues, poor quality of service, disparities, and demographic changes [23].

A Delphi study was conducted to establish a foundational understanding of the existing care landscape [24]. This study aimed to identify the current state of care provision, the desired future scenario, and the existing gaps in service delivery. Using a structured, iterative process, a panel of 223 experts from European countries Greece and Spain and non‐European countries of Egypt, Lebanon, and Tunisia, specialising in geriatric care, health policy, and social services, participated in successive rounds of anonymous consultation. The study was informed by the contributions of various experts, professionals, caregivers, individuals involved in care provision, and key stakeholders, whose insights, based on their professional expertise and lived experiences, facilitated a consensus on key issues. The findings revealed key deficiencies, including fragmented care models, inadequate integration of health and social services, and limited person‐centred approaches. Opportunities for improvement were identified in the promotion of multidisciplinary collaboration, digital health solutions, and community‐based interventions. To bridge these gaps, experts proposed strategies such as improving coordination between healthcare and social services, strengthening professional training in geriatric care, and implementing policies that prioritise equity and accessibility in long‐term care [24].

### 3.2 Determining the Aim and Purpose of the Analysis

Building on these insights, the present study seeks to develop a novel framework for the care of older people dependent on them or those at risk of social exclusion in the Mediterranean region. This study aims to propose a more comprehensive, responsive, person‐centred, and sustainable framework that aligns with the identified needs and future expectations.

### 3.3 Definition, Attributes, and Use of the Concept

A recent integrative review presents the main initiatives currently shaping socio‐health care practices for older people in the Mediterranean basin [15]. These initiatives incorporate person‐centred, holistic, and interdisciplinary approaches that align with the nursing metaparadigm: person, environment, health, and nursing practice, while also integrating transversal values such as digital innovation, cultural responsiveness, and gender‐sensitive care. Collectively, these initiatives underscore the importance of ensuring that older people, their families, and caregivers are actively involved in care planning and decision making. Autonomy, social inclusion, and mental well‐being are recurring themes, reflecting a broad conceptualisation of health that extends beyond physical well‐being. The care environment in these frameworks is not limited to clinical settings but encompasses home models, community centres, and some digital sources, facilitating accessibility and continuity of care. Interdisciplinary collaboration is a defining feature, with most practices incorporating multi‐professional teams to address the complex health and social needs of older populations. While some models prioritise nursing‐led care, others advocate for greater coordination between healthcare professionals, social workers, and informal caregivers. Furthermore, training programmes for caregivers are increasingly recognised as essential, enhancing the competencies of both formal and informal care providers [15].

**FIGURE 1:**
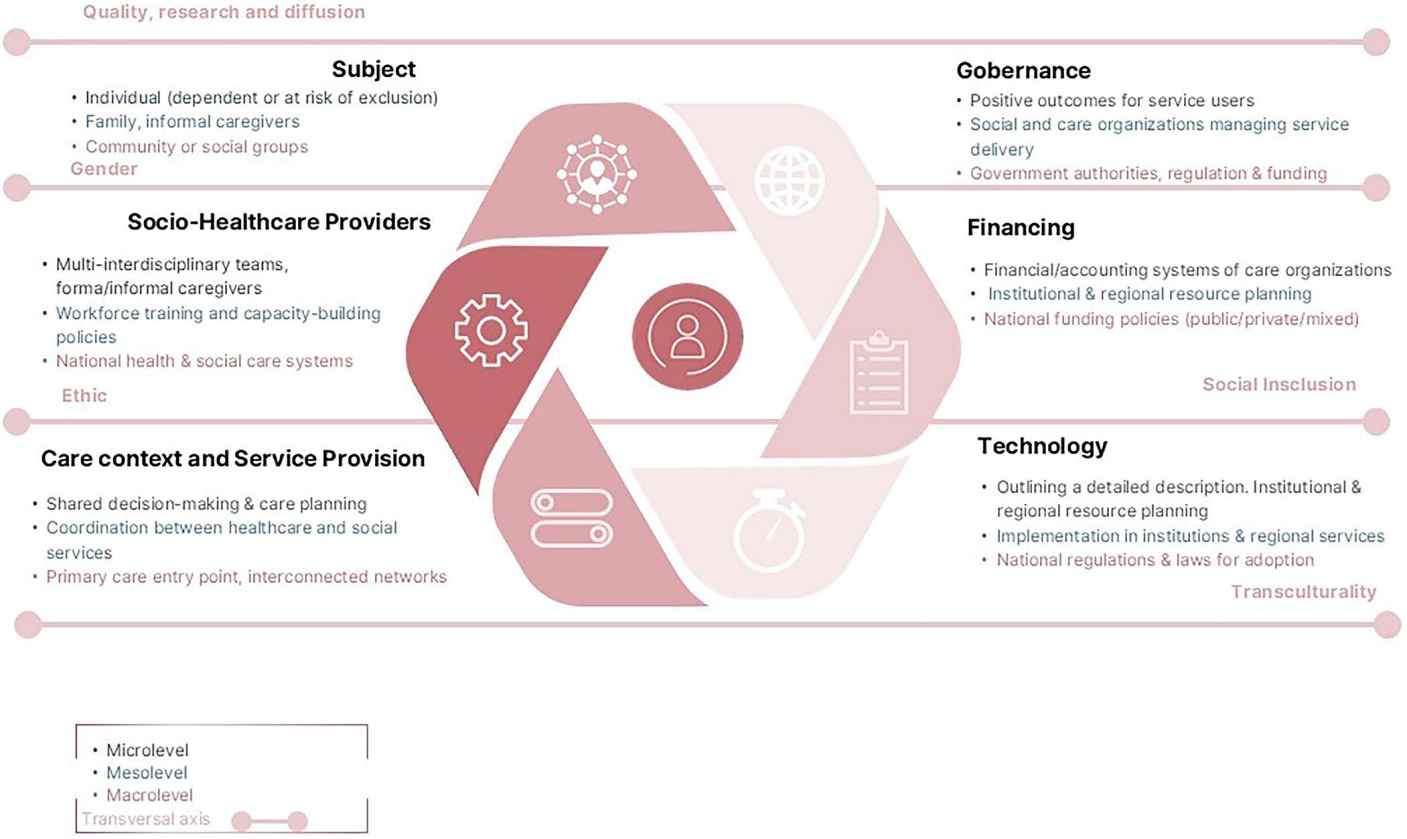
TEC‐MED theoretical framework and transversal concepts of the model.

Based on the former field work, the TEC‐MED framework was designed with six dimensions (person, socio‐health professionals, care context and service provision, technology, governance, and financing), and five transversal concepts (quality research, gender perspective, social inclusion, ethics, and transcultural approach). The considerations of these dimensions and concepts must be considered at micro, meso, and macro levels (Figure 1).

#### 3.3.1 Dimensions

##### 3.3.1.1 Person

The team conceptualised this dimension as the subject receiving socio‐health care, which may refer to the individual (micro level), the family (meso level), or community groups (macro level). The person must be placed at the center of the system, with their dependency care needs serving as the starting point of the care process. A holistic approach is required, considering not only their needs but also their preferences and socio‐economic context. Furthermore, it is essential to address cultural, ethical, gender‐related, and social equality and inclusion aspects.

##### 3.3.1.2 Socio‐Healthcare Providers

Socio‐healthcare providers comprise multi‐ and interdisciplinary teams that include healthcare and social professionals, as well as both formal and family‐based care (micro level). Policies and workforce planning must focus on capacity building and training for these care providers (meso and macro levels). Moreover, the role of caregivers, both formal and informal, must be recognized and valued, ensuring fair treatment and appropriate financial compensation. Caregivers are among the key actors in the provision of socio‐healthcare services, which must be of high quality, dignity, ethical, transcultural, and equitable.

##### 3.3.1.3 Care Context and Service Provision

Caring provided in socio‐health care refers to the act of delivering user‐centered services and constitutes the context in which care is provided to individuals. The ability to offer high‐quality and effective services is contingent upon the existence of an entry point into the system (macro level), which could be primary care. This primary level must, in turn, be horizontally and vertically coordinated with other healthcare levels and social care services (meso level). This network of interrelated services ensures integrated care for individuals, considering aspects of safety, efficiency, and continuity of care. Additionally, it is essential to involve individuals in the design and planning of their care, including shared decision‐making and planning at the micro level.

##### 3.3.1.4 Governance

Experts from the Lebanese team contributed to defining governance as the process by which social care organisations (meso level) ensure the effective provision of services. Governance promotes positive outcomes for service users (micro level) and encompasses a broad range of managerial and regulatory functions executed by governmental authorities (macro level). In this regard, governance is aimed at developing implementation and changing strategies tailored to the diverse care settings and contexts across Europe and the Mediterranean Basin. Good governance should be independent of political orientation, ensuring fair funding that provides universal protection for all citizens, regardless of their financial resources.

##### 3.3.1.5 Financing

Financing constitutes the economic support system and the foundation for developing any model. The available funding options include public, private, mixed, or non‐profit sources. This dimension encompasses financing priorities at the political and governmental levels (meso and macro), as well as the financial and accounting systems of the institutions and organisations delivering socio‐healthcare services (micro). Transparency and financial auditing serve as the cornerstone of this dimension, ensuring the precise delivery of socio‐healthcare services.

##### 3.3.1.6 Technology

This dimension encompasses various technological applications, including telemedicine, tracking technologies, data entry software, and artificial intelligence solutions for risk prediction. In the context of a care model, technology should enable rapid and integrated data processing while ensuring accessibility and ease of use for the target population (micro level). However, the successful adoption of technological innovations and solutions in developing a model depends on an integrated approach across different levels of management, beginning with regulations and established laws (macro level) and their effective national implementation (meso level).

#### 3.3.2 Transversal Values

##### 3.3.2.1 Quality Research

This value promotes the continuous assessment and refinement of care strategies, fostering an adaptive model that responds effectively to the evolving needs of older dependent individuals and those at risk of social exclusion. Research‐informed decision‐making enhances the sustainability and impact of interventions, facilitating the development of quality, innovative, and efficient evidence‐based care solutions that are designed to be sustainable over a long time.

##### 3.3.2.2 Gender Perspective

A gender‐sensitive approach recognizes the differential health and social care needs of older people based on gender‐related factors. The framework incorporates strategies aimed at reducing gender‐based disparities in care provision, recognizing the specific vulnerabilities of women as both care recipients and caregivers. By embedding gender equity principles into policy design, workforce development, and service delivery, the model strives to ensure inclusive and fair access to quality care for all individuals, regardless of gender identity.

##### 3.3.2.3 Social Inclusion

Social inclusion is fundamental to promoting equity and dignity within socio‐healthcare models. The framework prioritises the active engagement of older people in care planning and decision‐making, strengthening their sense of agency and autonomy. By fostering community‐based interventions, strengthening social networks, and improving accessibility to essential services, the model aims to mitigate the risks of isolation, discrimination, and marginalisation among vulnerable populations.

##### 3.3.2.4 Ethics

Ethical principles underpin every aspect of the framework, ensuring the protection of human dignity, autonomy, and rights. The model adheres to principles of justice, beneficence, and nonmaleficence, fostering a caring environment characterized by respect, transparency, and accountability. Ethical considerations extend to shared decision‐making, advanced care planning, and the equitable distribution of healthcare resources, aligned with both professional codes of conduct and broader human rights frameworks.

##### 3.3.2.5 Transcultural Approach

The framework recognizes the cultural diversity of the Mediterranean Basin and seeks to integrate transcultural perspectives into the care provided. Culturally responsive strategies address the specific beliefs, values, and needs of different ethnic and social groups, ensuring that healthcare services are inclusive and sensitive to the lived experiences of older individuals. By fostering intercultural dialogue and training healthcare professionals in culturally competent care, the model enhances trust, accessibility, and effectiveness in service delivery.

### 3.4 Case Studies

#### 3.4.1 Model Case

Ann, an 82‐year‐old woman, receives integrated socio‐healthcare services in accordance with the proposed TEC‐MED model framework. A retired artisan living alone in a small town, she has multiple chronic conditions, including diabetes and early‐stage dementia. Her care is managed by a multidisciplinary team that implements evidence‐based interventions such as structured medication management, cognitive stimulation therapy, or nutritional counselling.

At the micro level, Ann's preferences and values are respected in decision‐making, promoting her autonomy. Her decision to stay home is respected by carefully monitoring their health management and quality of life. Her care plan includes telemedicine for health monitoring, home visits from a community nurse, and social inclusion programmes. At the meso level, she has a strong support network of family and neighbours who assist with daily activities, reinforcing social cohesion. Gender‐sensitive policies acknowledge her past role as an unpaid caregiver, providing her with psychological support. At the macro level, her care is backed by government policies prioritising integrated services for older adults, implemented at the municipal level. Her pension, along with public funding, ensures financial sustainability. Ethical considerations guide her shared decision‐making process, empowering her to remain actively involved in her care. In addition, her cultural background is considered in interventions, ensuring inclusion. This multi‐level approach improves Ann's well‐being while aligning with evidence‐based elderly care practices.

#### 3.4.2 Borderline Case

Helen, a 79‐year‐old woman, experiences gender‐based disparities in access to care. Living with limited mobility after a stroke, she relies on her daughter, who provides unpaid care. While she has access to public healthcare services, no formal support is provided for her daughter, who struggles to balance caregiving with employment. The transversal values of gender equity and social inclusion in the framework are not fully applied, as the unpaid caregiving burden disproportionately affects women.

Furthermore, the principles of dignity and autonomy are not adequately upheld, since Helen is not actively involved in decisions regarding her long‐term care. Advanced care planning is not considered, restricting her ability to express her preferences for future health and social support. Similarly, the value of sustainability is not fully integrated, as the lack of structured caregiver support creates an unsustainable burden on informal care networks.

Although some ethical and clinical aspects align with the framework, incomplete application of transversal values, including gender equity, social inclusion, dignity, autonomy, and sustainability, hinders full implementation. Addressing these gaps would improve Helen's well‐being and ensure a more equitable and person‐centred approach to her care.

#### 3.4.3 Contrary Case

Serge, an 80‐year‐old man, is entirely excluded from socio‐healthcare services. A former agricultural worker with no formal pension, he lives in extreme poverty in a remote rural village. He has severe mobility impairments and relies on occasional help from neighbours. However, he does not have access to formal health care services due to financial and geographical barriers. His health deteriorates without medical intervention, and his social exclusion prevents him from participating in community activities.

In addition to these challenges, Serge faces significant transcultural barriers. He speaks a regional dialect that is not widely understood by healthcare providers, further complicating his ability to seek and receive medical care. The absence of culturally sensitive services exacerbates his isolation, limiting his ability to communicate his health concerns effectively.

There is no governance structure in place to ensure his inclusion in the healthcare system, and no ethical considerations are applied to support his right to dignified care. Serge's case represents a complete failure to implement the principles of the new framework, highlighting the urgent need for policies that ensure equitable and culturally inclusive access to social health services for vulnerable populations.

### 3.5 Antecedents and Consequences

The antecedents of the TEC‐MED framework are rooted in the pressing challenges and existing conditions within the Mediterranean basin. This region is experiencing a significant demographic shift characterised by an ageing population and a corresponding increase in chronic diseases and dependency among older individuals [25, 26]. This epidemiological trend has strained existing sociohealthcare systems, exposing critical gaps in service provision. A major problem is the fragmentation of social and healthcare services, which often operate in silos and fail to provide coordinated person‐centred care [27]. This lack of integration has limited the ability of current models to address the multifaceted needs of older adults, particularly those at risk of social exclusion.

Socioeconomic and cultural disparities further exacerbate these challenges. Inequities in access to care persist due to factors such as poverty, geographic isolation, and insufficient consideration of cultural diversity in care practices [28]. These disparities disproportionately affect vulnerable populations, deepening their marginalisation. Furthermore, a deficiency in cohesive policies and strategic frameworks aimed at uniting social and healthcare systems is noted [20]. Without such frameworks, efforts to establish holistic, accessible, and inclusive care models remain fragmented and inconsistent.

Implementing the TEC‐MED model framework anticipates bringing about transformative outcomes in socio‐health care systems across the Mediterranean region. A key consequence is the improvement of the quality of care through integrated and personalised approaches. By focussing on holistic health, the framework aims to improve the physical, emotional, and social well‐being of older adults, promoting better health outcomes and quality of life [29, 30]. Furthermore, the framework emphasises interdisciplinary collaboration, encouraging effective coordination between healthcare professionals, social workers, and caregivers.

Another significant consequence is the reduction of social inequalities. By addressing barriers to access to care, particularly for economically disadvantaged and geographically isolated populations, the TEC‐MED model framework promotes greater inclusivity and equity. The model design also prioritizes the empowerment of care recipients and their caregivers. Older people are actively involved in decision‐making processes, fostering autonomy and engagement, while caregivers benefit from enhanced recognition and support, reducing their burden and improving their well‐being [31, 32, 33].

In addition, the framework is expected to contribute to the sustainability of sociohealthcare systems. Through integrated care models, resource utilisation can be optimised, redundancies minimised, and cost efficiency improved. This systemic improvement supports long‐term sustainability, ensuring that care systems remain resilient and responsive to future demands [34, 35].

### 3.6 Empirical Referents

Empirical referents serve as measurable indicators that validate the existence and practical application of the TEC‐MED model framework and should provide tangible means to assess the integration of sociohealthcare services and their impact on dependent older adults and those at risk of social exclusion in the Mediterranean region.

One key empirical reference is the degree of service integration and interdisciplinary collaboration within socio‐healthcare settings. This can be measured through indicators such as the implementation of coordinated care pathways, the degree of collaboration between healthcare professionals and social workers, and the presence of multidisciplinary teams at the micro, meso, and macro levels. Quantitative data on referral rates between health and social services, as well as qualitative assessments from practitioners and service users, can offer information on the effectiveness of integration [36, 37, 38].

Another critical factor is the extent to which the principles of person‐centred care are embedded in practice. This can be evaluated through patient‐reported outcomes, including perceived autonomy in decision‐making, satisfaction with care, and access to holistic support services that address medical, psychological, and social needs [39, 40]. The presence of caring plans that explicitly incorporate shared decision making and advance care planning further substantiates the operationalisation of this principle at different levels, ensuring personalised care at the micro level, effective care networks at the meso level, and supportive policy frameworks at the macro level.

The commitment of the framework to equity and social inclusion can be empirically evaluated by examining disparities in access and utilisation of healthcare among vulnerable populations [41]. Data on the acceptance of services by older adults in rural or economically disadvantaged areas, as well as the availability of culturally tailored interventions for migrant and minority groups, serve as concrete indicators of the effectiveness of the model to promote inclusivity [42, 43].

The role of technology as a facilitator of integrated care is a fundamental determinant at the micro, meso, and macro levels. The adoption of telemedicine, digital health records, and AI‐driven predictive tools can be measured through system usage rates, patient adherence to digital solutions, and reductions in avoidable hospital admissions. Furthermore, qualitative feedback from older individuals on the usability and accessibility of digital tools offers a nuanced understanding of the technological dimension of the framework [42, 44, 45].

Another empirical reference refers to the empowerment and recognition of caregivers, both formal and informal. Metrics such as participation rates in caregiver training, the establishment of financial or psychosocial support schemes, and satisfaction surveys among caregivers provide measurable indicators of the responsiveness of the framework to their needs at all three levels, ensuring individual support (micro), network development (meso), and structural recognition of policies (macro) [44, 45, 46].

Finally, the sustainability and economic feasibility of the TEC‐MED model can be evaluated through effectiveness, for example on the increase of quality of life of older persons and caregivers or increasing the social and family support, and cost‐effectiveness analyses, assessing whether integrated socio‐healthcare delivery leads to reduced healthcare expenditures, optimised resource allocation, and improved long‐term financial sustainability [47, 48, 49]. Sustainability can be analysed at the microlevel through efficient use of resources in individual care, at the meso level through regional service coordination, and at the macrolevel through national policy and funding allocation.

## 4 Discussion

This study provides a comprehensive analysis of the concept of integrated socio‐health care for dependent older people and those at risk of social exclusion, as a process to establish the novel TEC‐MED care framework. Specifically, it examines its attributes, model and contrary cases, antecedents, consequences, and empirical references.

The definition of the TEC‐MED care model is supported by existing literature that reveals fragmented socio‐health care models, which particularly affect the older population [50]. Consequently, the WHO advocates for developing new approaches based on integrated care [51]. This fragmentation impacts the management of older people's complex needs [52], especially those with vulnerabilities such as chronic conditions, lack of autonomy, or risk of social exclusion due to factors like isolation, residing in remote areas, or gender inequalities. In particular, although women have a longer life expectancy, they often experience a poorer quality of life [53]. Inadequate socio‐health care services affect not only individuals but also system efficiency [27].

To address these issues, intensive fieldwork has been conducted to define a new model of care [15, 23, 24]. The findings underscore the importance of adopting an integrated, person‐centered social and health framework that responds sustainably and equitably to the complex needs of dependent older people at risk of social exclusion, as well as their families, particularly informal caregivers [11]. Although the TEC‐MED framework is interdisciplinary, the nursing metaparadigm, health, person, practice, and environment, plays a crucial role in the design of the model, serving as a reference to develop holistic, patient‐centred, and adaptable care frameworks [54]. The TEC‐MED model aims to improve health outcomes for older people from a broad, holistic perspective, focusing on health equity, autonomy in self‐care, mental health, well‐being, and family and community health and participation [15].

Conventional integrated care frameworks tend to emphasise clinical coordination or sector‐specific strategies, but often neglect the ethical, cultural, and social determinants that shape health outcomes [55]. The TECMED model remedies these limitations by articulating six interrelated dimensions and five cross‐cutting values that weave ethical principles, cultural responsiveness, and social inclusion into care planning and delivery. At the micro level, it supports personalised, person‐centred interventions that respect individual preferences. At the meso level, it promotes structured coordination between health and social services, underpinned by workforce training and institutional partnerships. At the macro level, it advocates inclusive policies, sustainable financing, and regulatory frameworks that assure equitable access. By integrating these elements, the model enhances continuity, accessibility, and responsiveness, particularly for vulnerable groups who are often overlooked by conventional approaches. This comprehensive orientation aligns with evidence that integrated care must move beyond process improvement to address the root causes of health inequities and to coordinate care across disciplines and settings [11].

The person is a key dimension of the TEC‐MED model, considered from a broad perspective that respects individual and family preferences and engages community resources by incorporating person‐, family‐, and community‐centred care approaches [56]. Within this dimension, the family/informal caregiver is an essential pillar for long‐term care of older individuals [57]. In summary, primary care recipients of the TEC‐MED model are older individuals and their caregivers; however, extended family and community, especially as sources of social support and health assets, are also subjects of care within a specific socioeconomic context.

Although integrated care is increasingly prioritized in ageing‐related policies, many existing frameworks remain limited to clinical coordination or sector‐specific strategies, often neglecting ethical, cultural, and social dimensions [52, 58]. In contrast, the TEC‐MED framework introduces a structurally integrated and ethically grounded approach. Its six dimensions and five transversal values enable multilevel coordination while embedding person‐, family‐, and community‐centered care. Furthermore, it leverages digital technologies to support accessibility and continuity, especially for socioeconomically and geographically vulnerable populations. The incorporation of social inclusion, gender equity, and transcultural responsiveness distinguishes TEC‐MED as an innovative and sustainable model for integrated socio‐healthcare in complex settings [30, 59].

Regarding practice, the TEC‐MED model includes sociohealth providers who should work in multidisciplinary teams that coordinate care through collaborative pathways, also considering patient and family participation. Informal caregivers are highlighted in this dimension, as they play a key role not only as care recipients but also as care providers, supported by professional care teams. Concerning the environment, the care context and the provision of services must have a user‐centred design. In particular, developing specialised training for workers to provide dignified, ethical, and evidence‐based care, as well as service provision monitoring [60]. Home care and primary care as entry points into the sociohealth system are crucial in the care environment, as supported by previous literature and framework development participants [11, 15, 24].

Although technology was not initially considered in Fawcett's metaparadigm, it has recently been recognised as a relevant aspect in developing theoretical frameworks, improving their effectiveness in contemporary healthcare settings [61]. In the TEC‐MED model, technological applications include telemedicine, tracking technologies, data entry software, and artificial intelligence solutions for predicting risk or improving system accessibility, especially for people living in remote areas. The TEC‐MED platform, which integrates standardised nursing languages into the decision‐making process, is developed within this context [19].

The final two dimensions of the TEC‐MED model focus on adopting and maintaining innovative, evidence‐based care solutions: financing and governance. Considering previous research, the model supports the welfare state; however, different funding options in each context must be considered, including public, private, mixed, or nonprofit sources. Transparency and financial auditing are cornerstones of this dimension, which, together with policy initiatives, contribute to the sustainability of the care model and provide universal protection for all citizens, regardless of their financial resources [34, 35].

Finally, relevant transversal aspects emerged during the development of the TEC‐MED model, as observed in previous work. These include cultural and ethical inclusion [59], integrating culturally tailored approaches that improve accessibility and equity, while ethical principles ensure dignity and autonomy [12]. The model also considers the gender perspective to address both the burden on women in informal care [62] and the impact of women's longer life expectancy, which places them at greater risk of vulnerability, such as loneliness [63]. Finally, adopting research‐informed decision‐making is crucial to developing quality initiatives and best practices in care [64].

### 4.1 Limitations and Strengths

The main limitation of this analysis is that it does not represent the entire spectrum of Mediterranean countries, as only six of the 14 potential countries participated in the TEC‐MED project. Furthermore, since this study is based on qualitative techniques, it is recommended to validate the model by transforming it into cost‐effective solutions through a pilot. In addition, this analysis did not assess the effectiveness, acceptability, or readiness of the TEC‐MED framework, as these aspects require subsequent empirical studies. The present work is limited to a theoretical clarification and conceptual grounding of the model, which constitutes a necessary preliminary step before testing its practical applicability.

A notable strength of the analysis is that it is grounded not only in a literature review, but also in fieldwork with relevant stakeholders from the six European and non‐European participant countries.

### 4.2 Implications for Practice

Adopting a person‐centred model based on the nursing metaparadigm, which covers a wide population, has significant implications for professional training, clinical practice, and leadership in public policy. On the one hand, it requires the implementation of continuous training programmes that equip professionals with communication, coordination, and culturally competent care skills [65]. On the other hand, training in the use of digital technologies, the management of complex cases, and educational programmes aimed at both formal and informal caregivers in clinical practice facilitate long‐term care tailored to the needs of older adults who are dependent and/or at risk of social exclusion [66, 67].

Furthermore, as nursing professionals become aware of their role in political participation as an expression of the sociopolitical knowledge pattern, their involvement improves the public policy process, presenting an opportunity for change given their strategic position as key figures in patient care within healthcare systems [68]. Within this model, the role of nurses serves as the driving force for transforming long‐term care and implementing public policies in healthcare systems.

This contribution also highlights the need to humanise nursing care by integrating relational, contextual, and care‐provision dimensions. Beyond interpersonal interactions, humanisation requires adapting care environments to uphold dignity and well‐being while fostering an organisational culture centred on respect, empathy, and personalised care [69]. By placing individuals at the core of clinical practice and health policies, this approach ensures care that is not only technically proficient but also ethically sound and socially just, ultimately enhancing both patient outcomes and professional satisfaction.

The TEC‐MED framework offers tangible benefits for older adults at risk of dependency or social exclusion. By integrating socio‐health services and tailoring interventions across the micro, meso, and macro levels, the model addresses key challenges faced by these populations, such as limited autonomy, lack of access to coordinated care, and marginalisation. Specifically, the inclusion of culturally responsive strategies, the recognition of informal caregivers, and the use of digital health solutions enhance accessibility, participation, and continuity of care. These features not only promote dignity and well‐being, but also help reduce health and social inequalities, making the model particularly valuable for the most vulnerable older adults in the Mediterranean region.

While this study provides a robust conceptual foundation for the TEC‐MED framework through formal concept analysis, further empirical research will be necessary to validate its effectiveness and applicability in real‐world socio‐healthcare settings. This theoretical grounding represents a critical first step toward future implementation and evaluation. In addition, the TEC‐MED model has been specifically developed for older adults with dependency or at risk of social exclusion. Its integrated, person‐centred, and multilevel structure could potentially be adapted to address the needs of other vulnerable primary care populations, such as individuals with multimorbidity who are not yet dependent but require complex care coordination. Future studies could explore the applicability of the model in such contexts.

## 5 Conclusion

The implementation of the TEC‐MED framework represents a strategic opportunity to transform social and healthcare systems in the Mediterranean Basin. Its person‐centred approach, technological integration, and focus on social determinants of health enable an effective response to the current and future challenges of caring for dependent older adults. However, the sustainability of this model will largely depend on political will, adequate funding, and the adoption of inclusive public policies that ensure equity and accessibility to services for all older adults in the region.

## Author Contributions

A.‐M.P.‐G., R.A.‐C. and M.L.‐S. conceptualised and drafted the manuscript; A.‐M.P.‐G., R.A.‐C. and M.L.‐S. conducted the data analysis; R.A.‐C. and A.‐M.P.‐G. designed and critically reviewed the manuscript, which M.L.‐S. also reviewed. All of the authors read and approved the final version of the manuscript.

## Ethics Statement

The study has received the ethical approval of the Research Ethics Committee of the Junta de Andalucía in Spain, given that the consortium's coordinating organization is the University of Seville in Spain (Reference no.: 2412‐N‐19).

## Consent

The authors have nothing to report.

## Conflicts of Interest

The authors declare no conflicts of interest.

## Data Availability

The data is available upon request to the corresponding author: Dr. Regina Allande‐Cussó, e‐mail: rallande@us.es.
